# Endocrine and haemodynamic changes in resistant hypertension, and blood pressure responses to spironolactone or amiloride: the PATHWAY-2 mechanisms substudies

**DOI:** 10.1016/S2213-8587(18)30071-8

**Published:** 2018-06

**Authors:** Bryan Williams, Thomas M MacDonald, Steve V Morant, David J Webb, Peter Sever, Gordon T McInnes, Ian Ford, J Kennedy Cruickshank, Mark J Caulfield, Sandosh Padmanabhan, Isla S Mackenzie, Jackie Salsbury, Morris J Brown, The British Hypertension Society programme of Prevention And Treatment of Hypertension With Algorithm based Therapy (PATHWAY) Study Group

**Affiliations:** a UCL Institute of Cardiovascular Sciences, University College London, London, UK; b National Institute for Health Research, UCL Hospitals Biomedical Research Centre, London, UK; c Medicines Monitoring Unit, Molecular and Clinical Medicine, University of Dundee, Dundee, UK; d Clinical Pharmacology Unit, Centre for Cardiovascular Science, Queen's Medical Research Institute, University of Edinburgh, Edinburgh, UK; e Centre of Circulatory Health, Imperial College London, London, UK; f BHF Glasgow Cardiovascular Research Centre, Institute of Cardiovascular and Medical Sciences, University of Glasgow, Glasgow, UK; g Robertson Centre for Biostatistics, University of Glasgow, Glasgow, UK; h Department of Nutritional Sciences, King's College London, London, UK; i William Harvey Research Institute, Queen Mary University of London, London, UK; j NIHR Barts Hospital Biomedical Research Centre, London, UK

## Abstract

**Background:**

In the PATHWAY-2 study of resistant hypertension, spironolactone reduced blood pressure substantially more than conventional antihypertensive drugs. We did three substudies to assess the mechanisms underlying this superiority and the pathogenesis of resistant hypertension.

**Methods:**

PATHWAY-2 was a randomised, double-blind crossover trial done at 14 UK primary and secondary care sites in 314 patients with resistant hypertension. Patients were given 12 weeks of once daily treatment with each of placebo, spironolactone 25–50 mg, bisoprolol 5–10 mg, and doxazosin 4–8 mg and the change in home systolic blood pressure was assessed as the primary outcome. In our three substudies, we assessed plasma aldosterone, renin, and aldosterone-to-renin ratio (ARR) as predictors of home systolic blood pressure, and estimated prevalence of primary aldosteronism (substudy 1); assessed the effects of each drug in terms of thoracic fluid index, cardiac index, stroke index, and systemic vascular resistance at seven sites with haemodynamic monitoring facilities (substudy 2); and assessed the effect of amiloride 10–20 mg once daily on clinic systolic blood pressure during an optional 6–12 week open-label runout phase (substudy 3). The PATHWAY-2 trial is registered with EudraCT, number 2008–007149–30, and ClinicalTrials.gov, number NCT02369081.

**Findings:**

Of the 314 patients in PATHWAY-2, 269 participated in one or more of the three substudies: 126 in substudy 1, 226 in substudy 2, and 146 in substudy 3. Home systolic blood pressure reduction by spironolactone was predicted by ARR (*r*^2^=0·13, p<0·0001) and plasma renin (*r*^2^=0·11, p=0·00024). 42 patients had low renin concentrations (predefined as the lowest tertile of plasma renin), of which 31 had a plasma aldosterone concentration greater than the mean value for all 126 patients (250 pmol/L). Thus, 31 (25% [95% CI 17–33]) of 126 patients were deemed to have inappropriately high aldosterone concentrations. Thoracic fluid content was reduced by 6·8% from baseline (95% CI 4·0 to 8·8; p<0·0001) with spironolactone, but not other treatments. Amiloride (10 mg once daily) reduced clinic systolic blood pressure by 20·4 mm Hg (95% CI 18·3–22·5), compared with a reduction of 18·3 mm Hg (16·2–20·5) with spironolactone (25 mg once daily). No serious adverse events were recorded, and adverse symptoms were not systematically recorded after the end of the double-blind treatment. Mean plasma potassium concentrations increased from 4·02 mmol/L (95% CI 3·95–4·08) on placebo to 4·50 (4·44–4·57) on amiloride (p<0·0001).

**Interpretation:**

Our results suggest that resistant hypertension is commonly a salt-retaining state, most likely due to inappropriate aldosterone secretion. Mineralocorticoid receptor blockade by spironolactone overcomes the salt retention and resistance of hypertension to treatment. Amiloride seems to be as effective an antihypertensive as spironolactone, offering a substitute treatment for resistant hypertension.

**Funding:**

British Heart Foundation and UK National Institute for Health Research.

## Introduction

Resistant hypertension is defined as a blood pressure that is uncontrolled despite treatment with at least three blood pressure-lowering drugs, including a diuretic, usually also including an angiotensin-converting enzyme (ACE) inhibitor or angiotensin receptor blocker (ARB) and a calcium channel blocker (CCB), and after exclusion of treatable secondary causes of hypertension.^1^ Resistant hypertension affects up to 10% of patients treated for hypertension and is associated with a high risk of cardiovascular morbidity and mortality.^2^ In the randomised, placebo-controlled crossover trial PATHWAY-2,^3^ we tested the recommendation^4^ to treat resistant hypertension by addition of a drug that blocks either the mineralocorticoid or adrenergic receptors. In PATHWAY-2, the mineralocorticoid receptor antagonist spironolactone (25–50 mg per day) was more effective at lowering blood pressure than bisoprolol (5–20 mg daily), doxazosin (4–8 mg daily), or placebo.^3^ This finding is supported by evidence from observational studies.^5^

**Research in context**

**Evidence before this study**

We previously reported the results of the PATHWAY-2 randomised controlled trial, which showed that low-dose spironolactone (25–50 mg daily), when added to standard blood pressure-lowering drugs, was substantially more effective at lowering blood pressure in patients with resistant hypertension than placebo or alternative blood pressure-lowering drugs (bisoprolol or doxazosin). On Sept 17, 2017, we searched MEDLINE, Embase, and the Cochrane Central Register of Controlled Trials using the search terms “resistant hypertension”, “pathophysiology”, “mechanisms”, “amiloride”, “hemodynamics”, and “aldosterone” for reports in English published up to July 31, 2017. Our search strategy included reports of randomised controlled trials as well as open and observational studies of drug treatment of resistant hypertension that included any data analysing mechanisms and pathophysiology of resistant hypertension or the use of amiloride. The available evidence was scarce. Findings from two observational studies had suggested that plasma renin concentrations were often more suppressed than anticipated in patients with resistant hypertension, consistent with this being a sodium-retaining and volume-expanded state. In another study, non-invasive haemodynamic measurements via impedance cardiography were used to establish whether treatment adjusted on the basis of haemodynamic characteristics would be more effective than empirical treatment at lowering blood pressure in resistant hypertension, but the findings were inconclusive. In a prospective study of 88 consecutive patients referred to a university clinic for resistant hypertension, researchers reported that 18 (20%) had increased urinary aldosterone and suppressed plasma renin concentrations, despite salt intake in excess of 200 mmol/24 h. Background treatment, including β-blockade, was not discontinued. Authors of other observational studies and commentaries have speculated that many cases of resistant hypertension might result from undetected aldosterone-producing adenomas. Results of many studies have shown the efficacy of low-dose (2·5–5·0 mg) amiloride added to thiazide, including reduction of morbidity and mortality. We have previously reported the efficacy of high-dose (10–40 mg) amiloride in treated hypertension, but amiloride has not previously been compared with spironolactone or other drugs in patients with resistant hypertension.

**Added value of this study**

In the PATHWAY-2 trial, spironolactone reduced blood pressure substantially more than conventional antihypertensive drugs in patients with resistant hypertension. In order for the results of PATHWAY-2 to change practice, it was important to show a mechanistic basis underpinning the superiority of spironolactone. The results of the PATHWAY-2 mechanisms substudies show that the efficacy of spironolactone as an antihypertensive drug could be anticipated from our findings that resistant hypertension is a salt-retaining condition, associated with high aldosterone-to-renin ratios, and that spironolactone was substantially superior to the other standard antihypertensive drugs in reducing indices of salt and water retention—ie, it was reducing blood pressure primarily via diuretic actions. Physicians who are hesitant to prescribe spironolactone for hypertension, either because it is not a universally licensed indication, or because of antiandrogen-related intolerance, will be reassured by our additional finding that amiloride 10–20 mg achieved similar reductions in blood pressure as spironolactone, with similar, slight changes in electrolytes.

**Implications of all the available evidence**

We propose that spironolactone, or amiloride if spironolactone is not tolerated, should be first-line treatment for resistant hypertension, in addition to background treatment with an angiotensin-converting enzyme inhibitor or angiotensin receptor blocker, a calcium channel blocker, and a diuretic, in patients with an eGFR greater than 45 mL/min/1·73m^2^ and serum potassium within the normal range. Among this cohort are likely to be some —perhaps many—patients whose hypertension is caused by primary aldosteronism. We encourage a reconsideration of which diagnostic thresholds for primary aldosteronism are appropriate in patients with resistant hypertension, to facilitate recognition of a potentially curable aldosterone-producing adenomas in patients with resistant hypertension.

Understanding the mechanism of the blood pressure-lowering superiority of spironolactone in resistant hypertension would help to delineate the pathophysiological basis of resistant hypertension and provide a rationale for developing alternative treatment strategies for patients in whom spironolactone is poorly tolerated. The hypothesis underpinning PATHWAY-2 was that resistant hypertension is predominantly a sodium-retaining state (despite background treatment with thiazide-type diuretics) and that further diuretic (more correctly, natriuretic) treatment would be the most effective means of lowering blood pressure.^3^ PATHWAY-2 incorporated a series of prespecified hormonal and haemodynamic measurements designed to facilitate investigation of the pathophysiology of resistant hypertension and its drug treatment.^6^ We now report the results of these analyses, which address three clinically important questions.

First, what is the relation between baseline plasma renin, aldosterone, and the aldosterone-to-renin ratio (ARR) and the blood pressure-lowering response to spironolactone, bisoprolol, doxazosin, and placebo? Consistent with our hypothesis that resistant hypertension is predominantly a sodium-retaining state, we expected plasma renin to be relatively suppressed despite background treatment with an ACE inhibitor or an ARB, a CCB, and a diuretic—ie, the so-called A+C+D treatment strategy.^3^, ^4^ A finding of a suppressed renin concentration, despite treatments that usually increase plasma renin, would be consistent with resistant hypertension being a sodium-retaining state. We also hypothesised that the blood pressure-lowering response to spironolactone, but not to other drugs, would be greatest in participants with the lowest plasma renin concentrations and highest ARRs, consistent with sodium retention being largely a consequence of autonomous aldosterone production. The proportion of patients exceeding validated thresholds for the diagnosis of primary aldosteronism was also assessed.

Second, we aimed to assess the haemodynamic responses to the various drug treatments and placebo and their effect on cardiac output, systemic vascular resistance, and thoracic fluid content, testing the hypothesis that the superior action of spironolactone in lowering blood pressure in resistant hypertension would be consistent with those of a diuretic.

Third, we hypothesised that if the superiority of spironolactone in resistant hypertension was due to its natriuretic actions, then amiloride would similarly decrease blood pressure. Like spironolactone, amiloride is a distal tubular diuretic that inhibits the aldosterone-sensitive epithelial sodium channel. Notably, a low-dose thiazide and amiloride combination achieved a greater reduction in blood pressure from baseline than high-dose thiazide alone in patients with hypertension in the PATHWAY-3 trial.^7^

## Methods

### Study design and participants

PATHWAY-2 was a 12-month, double-blind, placebo-controlled, randomised, crossover trial done at 12 secondary care sites and two primary care sites in the UK, in patients aged 18–79 years with systolic blood pressure of at least 140 mm Hg and home systolic average blood pressure of at least 130 mm Hg despite treatment with maximum tolerated doses of three blood pressure-lowering drugs (ie, A+C+D). Secondary causes of hypertension had been excluded and specific procedures were done to confirm resistant hypertension and patient adherence to their baseline medications.^6^ In the final year of PATHWAY-2, a new mass spectrometric assay permitted a spot urine test at baseline and the end of each double-blind phase to be checked for background and study drugs.^8^

**Figure 1:**
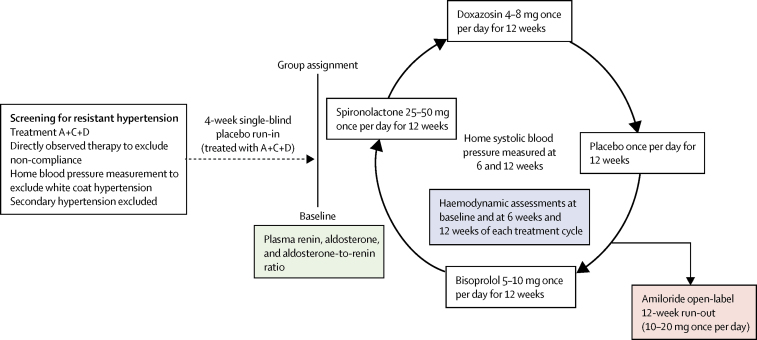
PATHWAY 2 study design Stages in the crossover trial at which measurements for the three mechanistic substudies were taken. Green shows substudy 1, blue shows substudy 2, and pink shows substudy 3. During the 12-week drug cycles, the lower dose was given for the first 6 weeks, then the higher dose was given for the second 6 weeks. No washout period was used between cycles. The figure shows one of the 24 possible passages through the drug cycles, the order of which was randomly assigned within blocks of 24 patients. A+C+D=an angiotensin-converting enzyme inhibitor or angiotensin receptor blocker, a calcium channel blocker, and a diuretic.

The haemodynamics and amiloride runout substudies were incorporated within the original protocol of PATHWAY-2, and the aldosterone substudy was added later. By design, each was done in a subset of patients, restricted by either recruitment date (aldosterone measurements [substudy 1]), setting (haemodynamic measurements [substudy 2]), or preference of the patient or doctor (amiloride runout [substudy 3]).^3^, ^6^ The overall design of PATHWAY-2 and its incorporated substudies is shown in figure 1. Patients from the intention-to-treat population in the main study were included in analysis of substudy 1 when measurements of aldosterone and renin were available; substudy 2 when measurements of haemodynamic parameters were available; and substudy 3 when clinic blood pressure readings after receiving at least one dose of amiloride were available.

In 2012, roughly halfway through recruitment for PATHWAY-2, additional funding permitted the addition of plasma aldosterone, and thus ARR, to the baseline measurements. The objective of substudy 1 was to compare the ability of plasma aldosterone, renin, and ARR to predict the blood pressure response to each drug and to explore the role of aldosterone in patients with resistant hypertension. Thus, patients recruited after 2012 with available baseline aldosterone measurements were included in substudy 1.

Patients were eligible for substudy 2 dependent on which location they were recruited from; seven of the 12 secondary care sites had the necessary specialist equipment. Substudy 2 assessed the effects of spironolactone, bisoprolol, and doxazosin treatments on thoracic fluid, cardiac index, and systemic vascular resistance and whether patients' most effective drug was predicted by increased baseline measurements of the parameter most likely to be reduced by that drug.

After the double-blind rotation, patients were offered the option of returning to primary care or continuing into an open-label runout phase (substudy 3). This opportunity for a non-randomised study of an alternative to spironolactone arose from the need for a period between each patient's final visit and completion of their electronic record, after which their best treatment could be unmasked. In this open-label phase, we assessed whether amiloride would have similar superiority to the other study drugs as was hypothesised for spironolactone, and, if so, whether a correlation between responses would support switching from spironolactone to amiloride in patients who became intolerant of spironolactone.

All study participants provided written informed consent as part of consent for the main trial and the protocol for these studies was approved by the Cambridge South Research Ethics Committee.

### Procedures

In the main PATHWAY-2 trial, after a 4-week single-blind placebo run-in, patients rotated through four cycles of once-daily oral treatment with spironolactone 25–50 mg, doxazosin 4–8 mg, bisoprolol 5–10 mg, and placebo, each for 12 weeks with forced titration to the higher dose after 6 weeks. The order in which drugs were administered to each patient was randomly assigned via a central computer system. Patients and investigators were masked to drug treatment groups. Blood was collected at the end of the placebo run-in, and batched results did not inform eligibility of individual patients for either the main trial or its substudies. Plasma renin was measured in a central laboratory as direct renin mass using the LIAISON automated chemiluminescent immunoassay (DiaSorin, Saluggia, Italy).^9^ Plasma aldosterone was also measured using the LIAISON automated chemiluminescent immunoassay^10^ or by mass spectrometry.

For substudy 2, non-invasive haemodynamic assessments of fluid balance, cardiac performance, and vascular resistance were done with the patient supine, at baseline and at the end of each treatment cycle, using thoracic electrical bioimpedance cardiography (CardioDynamics BioZ Impedance Cardiography Hemodynamic Monitor, CardioDynamics, San Diego, CA, USA).^10^ Four pairs of electrocardiogram electrodes were applied to the base of the neck and the lower thorax at the diaphragm level, and a high frequency, low magnitude current was applied. The difference between input and sensed voltage is established by the impedance of the thorax, which is inversely proportional to thoracic fluid volume. Stroke volume is calculated from the change of impedance (thoracic fluid content) over the cardiac cycle time. Because cardiac output and total body fluid volume is related to body mass, all blood flow parameters were indexed to body surface area in m^2^. Stroke index was calculated as stroke volume divided by body surface area (mL per heart beat per m^2^). Cardiac index (cardiac output) was calculated as stroke index multiplied by heart rate (L/min/m^2^). Systemic vascular resistance index was then derived from the measurements of cardiac index and blood pressure (dyn ×    sec/cm⁵/m^2^). Thoracic fluid content index, an index of body fluid volume, is expressed as 1/k*Ω*/m^2^. Previous studies have shown these primary variables to be highly reproducible on repeated measures in the same patient, separated by days or weeks.^10^, ^11^

In substudy 3, participating patients received amiloride 10 mg once daily for 6 weeks, followed by the option to up-titrate to 20 mg daily for a further 6 weeks if blood pressure remained uncontrolled (figure 1). Seated clinic blood pressure was measured after 6 and 12 weeks of amiloride treatment.

In the main PATHWAY-2 study, blood pressure was measured both as a home blood pressure average and seated clinic blood pressure. For substudy 1 examining the relations between hormones and the blood pressure response to treatment, home systolic blood pressure measurements were used. Home blood pressure was a single mean of 24 seated home blood pressure readings recorded in the morning and evening for 4 consecutive days, with triplicate readings at each measurement session. Home blood pressure was recorded over the 4 days before the baseline visit at the end of placebo run-in, and before the 6-week and 12-week visits of each double-blind treatment cycle. If incomplete, a minimum of six of the 24 possible blood pressure recordings over 4 days was required, or patients were excluded from the analysis.

Home blood pressure was not recorded during the open-label amiloride runout phase. Thus in substudy 3, the clinic blood pressure-lowering effect of amiloride was compared with clinic blood pressure reduction on other active treatments or placebo. Clinic blood pressure was the average of the last two of the triplicate readings recorded at the 6-weekly study visits during both the double-blind treatment cycles and the open-label amiloride runout. Visits could be morning or afternoon, but were at a consistent time of day for each patient. The home and clinic blood pressures were measured by a WatchBP Home monitor (Microlife, Clearwater, FL, USA), allocated to the patient for the year of double-blind treatment.

### Outcomes

The prespecified objectives and outcome measures for these mechanistic substudies of the PATHWAY-2 trial were to define the relations between baseline plasma renin, aldosterone, and ARR (on background treatment with A+C+D) and the blood pressure response to spironolactone, doxazosin, bisoprolol, and placebo; to analyse the haemodynamic response to the blood pressure-lowering treatments in resistant hypertension to establish their most likely mechanism of action; and to use the open-label runout phase of the study to establish whether amiloride would achieve blood pressure reductions similar to spironolactone in patients with resistant hypertension. Exploratory analyses of the prevalence of primary aldosteronism were also done. Only serious adverse events were recorded during the open-label amiloride runout.

In the aldosterone substudy (substudy 1) of patients who had a baseline measurement of ARR, the main outcomes were the baseline measurement of ARR, and the regression upon this of the change from baseline in home systolic blood pressure averaged (mean) across the 6-week and 12-week visits of each double-blind treatment. In the haemodynamic substudy (substudy 2) of patients at the seven sites that had a CardioDynamics BioZ Impedance Cardiography Hemodynamic Monitor (SonoSite, San Diego, CA USA), the main outcomes were the changes in thoracic fluid index, cardiac index, stroke index, and systemic vascular resistance between baseline and the end of each double-blind treatment. In the amiloride substudy (substudy 3) of patients who continued after the double-blind treatment cycles into an optional open-label runout on amiloride, the main outcome was change in clinic systolic blood pressure between baseline and end of 6 weeks (low-dose) or 12 weeks (high-dose) of amiloride treatment. In all substudies, treatment refers to the study drug administered in addition to the background treatment with A+C+D (an ACE inhibitor or an ARB, a CCB, and a diuretic).

### Statistical analysis

This PATHWAY-2 mechanisms study is a series of prespecified substudies embedded within the PATHWAY-2 trial.^6^ The sample size and statistical power calculations were done for the primary outcome of the PATHWAY-2 trial and do not apply to these substudies.

In substudy 1, we used regression analyses to explore the relation between each of baseline plasma renin, aldosterone, and the ARR and blood pressure responses to the active treatments and placebo. Exploratory analyses were post hoc, but when appropriate followed the prespecified PATHWAY definition of high and low renin concentrations being those within the upper and lower tertiles of the plasma renin distribution. The prevalence of primary aldosteronism was estimated using the threshold of 60·94 for ARR in the post-captopril suppression test, and a threshold of 5·3 (mmol L^−1^)^−1^ for the SUSPPUP ratio ([serum sodium/urinary sodium])/([serum potassium^2^/urinary potassium]).^12^, ^13^ In substudy 2, for analysis of haemodynamic responses to drug treatment, and to assess them as predictors of blood pressure response, we used mixed-effects models with an unstructured covariance matrix for repeat observations in the same patient. In substudy 3, we also used mixed models for blood pressure response to amiloride versus other active treatments or placebo.

In the mixed models, patients were defined as random effects, with no structure imposed on the within-subject covariance matrix, and all other covariates were regarded as fixed. Analyses were done with SAS, version 9.3 (Cary, NC, USA). Data checking for compliance with protocol were done by the Robertson Centre for Biostatistics, University of Glasgow (UK). The PATHWAY-2 trial is registered with EudraCT, number 2008–007149–30, and ClinicalTrials.gov, number NCT02369081.

### Role of the funding source

The funders of the study had no role in study design, data collection, data analysis, data interpretation, or the writing of the report. The investigators and all authors had sole discretion in the data analysis, interpretation, writing of the report, and the decision to submit for publication. The corresponding author had full access to all of the data and the final responsibility to submit for publication.

## Results

**Figure 2:**
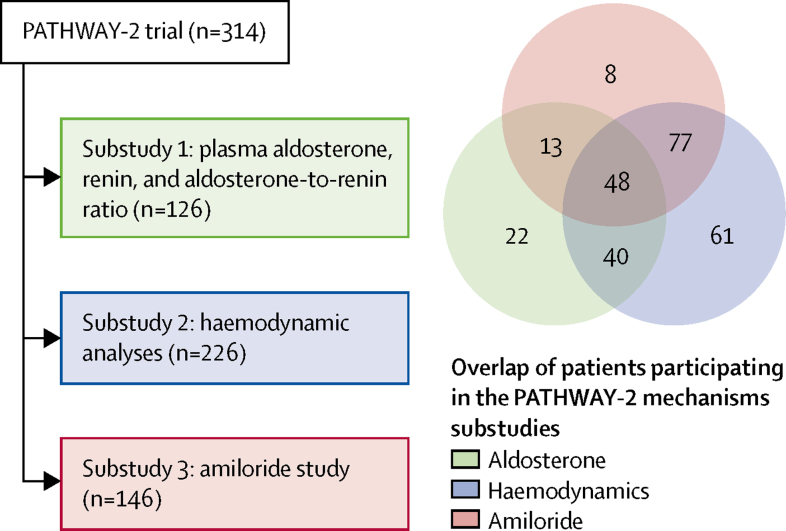
Participant numbers and measurements in the PATHWAY-2 mechanisms substudies

Between May 15, 2009, and July 8, 2014, we screened 436 patients for the PATHWAY-2 study. 335 were randomly assigned to treatment groups, of whom 21 had no follow-up for any drug and were excluded from the intention-to-treat analysis, which comprised 314 patients. Of these, 126 patients had baseline measurements of aldosterone and ARR, 226 participated in the haemodynamic analyses with impedance cardiography, and 146 participated in the amiloride runout phase of the study (figure 2).

The baseline characteristics of the patients in the three substudies and the overall trial population were similar (table). The mean baseline clinic blood pressure in the overall trial population was 157·4/90·3 mm Hg (SD 14·3/11·5); baseline electrolytes and estimated glomerular filtration rate (eGFR) were normal; and 24-h urinary sodium concentrations were about 150 mmol (equivalent to a salt intake of 9 g per day).

**Table:** Baseline characteristics of patients in the PATHWAY-2 trial and the PATHWAY-2 mechanisms substudies

	**Substudies**			**Main trial (n=314)**
	Aldosterone (n=126)	Haemodynamics (n=226)	Amiloride (n=146)	
Age, years	60·3 (9·6)	61·1 (9·5)	59·6 (10·1)	61·2 (9·6)
Men	86 (70%)	159 (70%)	109 (75%)	217 (69%)
Women	40 (30%)	67 (30%)	37 (25%)	97 (31%)
Bodyweight, kg	94·1 (17·6)	94·9 (19·0)	96·9 (20·3)	93·9 (18·3)
Systolic blood pressure, mm Hg	159·0 (14·3)	156·7 (14·2)	158·0 (14·0)	157·4 (14·3)
Diastolic blood pressure, mm Hg	92·4 (11·4)	89·9 (11·7)	91·0 (11·3)	90·3 (11·5)
Heart rate, beats per min	77·0 (12·3)	77·5 (11·9)	77·8 (11·1)	77·3 (12·2)
24 h urine Na^+^, mmol	151·6 (74·7)	148·5 (73·5)	153·0 (73·5)	138·1 (71·7)
24 h urine K^+^, mmol	79·0 (27·4)	72·5 (28·3)	77·4 (29·6)	70·8 (29·7)
Plasma Na^+^, mmol/L	139·2 (3·2)	139·7 (3·1)	140·1 (2·8)	139·6 (3·0)
Plasma K^+^, mmol/L	4·07 (0·44)	4·08 (0·46)	4·02 (0·41)	4·08 (0·44)
eGFR, mL/min/1·73m^2^	97·4 (26·6)	93·1 (26·6)	97·6 (25·5)	91·6 (26·8)
Diabetes	12 (10%)	39 (17%)	22 (15%)	43 (14%)
Renin, mU/L	33 (14·0–77·0)	33 (13·5–71·5)	34 (14·0–92·0)	34 (14·0–95·0)
Aldosterone, pmol/L	262 (179–352)	247 (178–343)	270 (187–353)	262 (179–352)

Data are mean (SD), n (%), or median (IQR). eGFR=estimated glomerular filtration rate.

**Figure 3:**
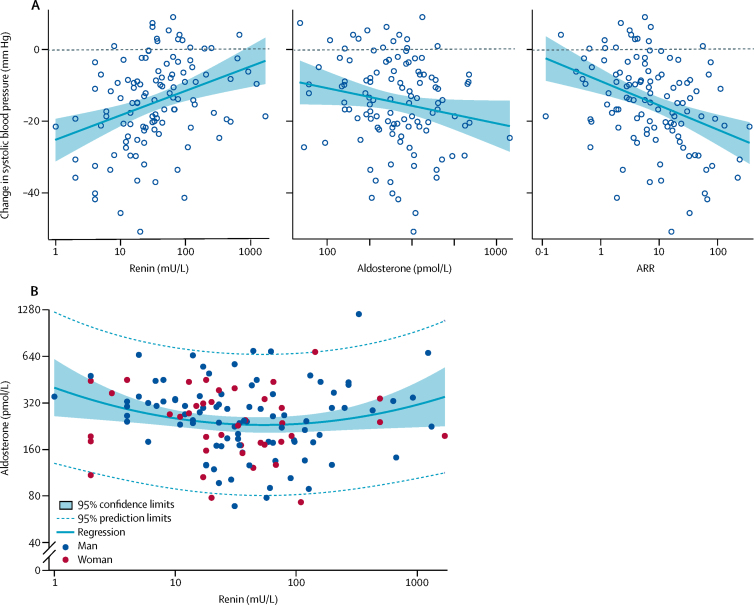
Correlations of plasma aldosterone, renin, and ARR, with blood pressure response to spironolactone averaged (mean) across the 6-week and 12-week visits of each treatment cycle (A) Relation between baseline plasma renin, aldosterone, and the ARR and the home systolic blood pressure response to spironolactone. (B) Best-fit relation between plasma aldosterone and renin concentrations at baseline. Regression equations for change in systolic blood pressure (*y*): *y*=(−25·20)+6·86 × (log_10_renin), *r*^2^=0·116 (proportion of variance accounted for by the model); *y*=8·92–9·85 × (log_10_aldosterone), *r*^2^=0·034; and *y*=(−8·87)–6·87 × (log_10_ARR), *r*^2^=0·138. Regression equation for aldosterone *vs* renin: log_10_aldosterone=2·60–0·279 × (log_10_renin) + 0·081 × (log_10_renin)^2^, *r*^2^=0·043. ARR=aldosterone-to-renin ratio.

In substudy 1, the blood pressure-lowering response to spironolactone was predicted, in order of significance, by baseline ARR (*r*^2^=0·13, p<0·0001), by plasma renin (*r*^2^=0·11, p=0·00024; greatest in patients whose plasma renin was suppressed), and weakly by plasma aldosterone alone (*r*^2^=0·025, p=0·052; figure 3A). No evident associations were identified between baseline plasma renin, aldosterone, or the ARR and the blood pressure response to placebo, doxazosin, or bisoprolol (appendix).

Aldosterone and renin concentrations were not linearly correlated (*r*^2^=0·0026, slope −0·019 [SE 0·032]). The best fit was quadratic (*r*^2^=0·043, p=0·060; figure 3B). 42 patients were in the lowest tertile of plasma renin concentration (renin <19 mU/L), of which 31 had a plasma aldosterone concentration greater than the mean for all 126 patients (250 pmol/L). Thus, a prevalence of 31 (25% [95% CI 17–33]) of 126 patients was inferred for inappropriately high aldosterone concentrations (ie, higher than average aldosterone in association with low plasma renin). Of the 42 patients in the highest tertile (renin >55 mU/L), 20 had a plasma aldosterone concentration greater than the mean. A post-hoc analysis by sex showed a significant quadratic association between aldosterone and renin concentrations in men (*r*^2^=0·090, p=0·016), but not in women (r^2^=0·005, p=0·87; appendix). Further exploration of the prevalence of primary aldosteronism in this cohort was prompted by the paucity of datapoints in the left lower quadrants of the graphs (figure 3B; appendix), together with the superiority of spironolactone versus conventional antihypertensive drugs in achieving target blood pressure in the main study and the prediction of blood pressure response by ARR. Lower and upper estimates for prevalence were reached using two published criteria. 13 (10% [95% CI 6–17]) of 126 patients, all of whom were receiving maximum tolerated doses of ACE inhibitor or ARB, had an ARR greater than 60·94 pmol/L^−1^ per mU/L, the recently validated threshold for post-captopril suppression.^12^ A less conservative estimate was obtained using the SUSPPUP index of sodium and potassium clearance: 48 (38%, 95% CI 33–43) of 126 patients had a value greater than 5·3 (mmol L^−1^)^−1^.^13^ Similar to ARR, SUSPPUP predicted home systolic blood pressure response to spironolactone, but not to the other trial drugs (appendix).

**Figure 4:**
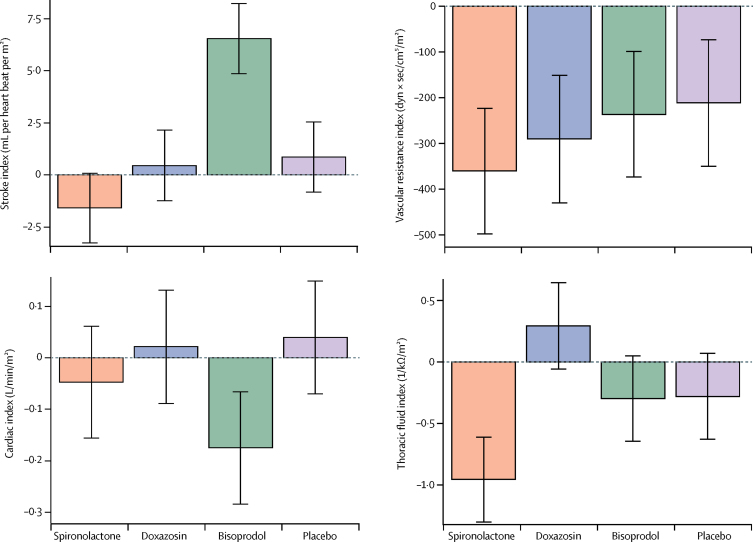
Change in haemodynamic parameters from baseline after 12 weeks treatment with spironolactone, doxazosin, bisoprolol, and placebo Stroke index, cardiac index, vascular resistance index, and thoracic fluid index were measured at baseline and the end of each double-blind treatment cycle. Least squares means adjusted for gender, height, weight, smoking history, baseline systolic blood pressure, and the baseline measurement of the outcome, from a mixed model allowing for correlations between repeat measurements in each patient. Coloured bars show least squares mean values and black bars show 95% CIs.

In substudy 2, the changes in cardiac output, systemic vascular resistance, and thoracic fluid volume from baseline to the end of the 12-week treatment cycle with each treatment were assessed (figure 4). Spironolactone significantly reduced thoracic fluid index by about 6·8% (95% CI 4·0 to 8·8), with a change of −1·0 1/kΩ/m^2^ (95% CI −1·3 to −0·6; p<0·0001). Thoracic fluid index was significantly increased by 0·57 1/kΩ/m^2^ with doxazosin compared with placebo (95% CI 0·26 to 0·89; p=0·00035; figure 4; appendix). Changes in thoracic fluid were associated with similar changes in bodyweight (appendix). Bisoprolol reduced cardiac index by 0·17 L/min/m^2^ (0·07–0·28; p=0·0018) and increased stroke volume index by 6·5 mL per heart beat per m^2^ (4·9–8·2; p<0·0001), reflecting a reduction in heart rate. Vascular resistance index showed a small but significant reduction with all treatments, including placebo, with no apparent differences between treatments (p=0·066). Baseline haemodynamic characteristics did not predict the blood pressure response to treatment (appendix).

**Figure 5:**
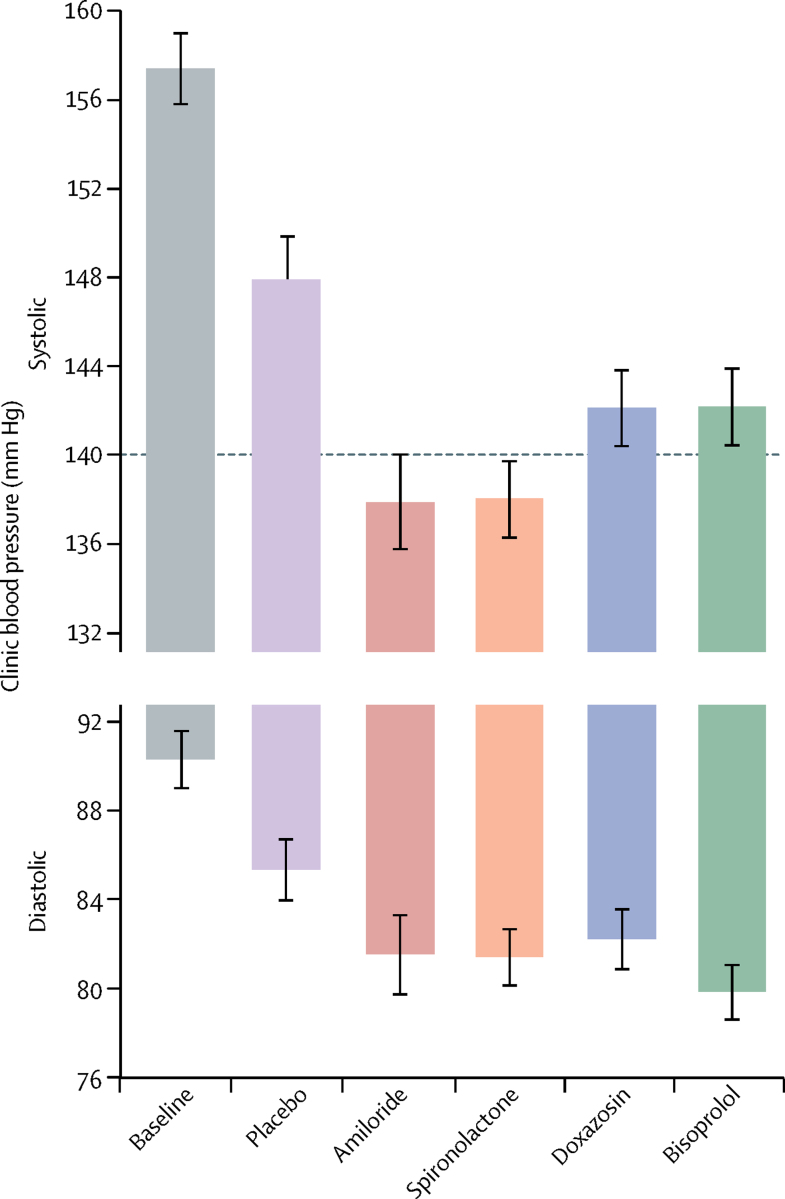
Effect of placebo, amiloride, spironolactone, doxazosin, and bisoprolol on clinic blood pressure after 6 weeks of treatment Both amiloride and spironolactone reduced systolic and diastolic blood pressure (unadjusted means) versus placebo (p<0·0001) and versus both doxazosin or bisoprolol (p<0·0001). The horizontal line at 140 mm Hg shows target clinic systolic blood pressure. Coloured bars show mean values and black bars show 95% CIs.

**Figure 6:**
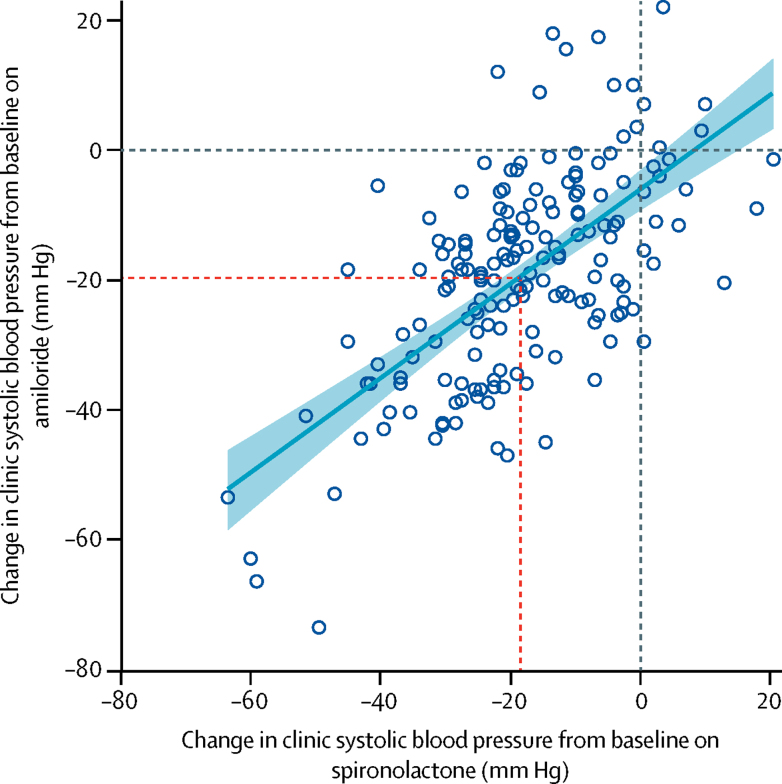
Correlation between change in systolic blood pressure in patients treated with amiloride and in those treated with spironolactone after 6 weeks of treatment Dotted lines show corresponding points on y axis (for amiloride) and x axis (for spironolactone) for mean responses (red) and zero responses (blue).

In the 144 patients who entered the amiloride runout phase (substudy 3), clinic blood pressure after 6 weeks was similar to the patients' previous measurements on spironolactone, and much lower than those on either placebo or the other two active treatments (p<0·0001; figure 5). The reductions in clinic systolic blood pressure from baseline to 6 weeks were 20·4 mm Hg (95% CI 18·3–22·5) with 10 mg amiloride and 18·3 mm Hg (16·2–20·5) with 25 mg spironolactone (appendix). The superiority of amiloride over other drugs and placebo was similar to that reported for spironolactone for all patients in the main trial. During the runout phase, no forced up-titration was done. In 47 patients whose blood pressure remained uncontrolled after 6 weeks of treatment with amiloride 10 mg daily and so were given 20 mg for the second 6 weeks, a similar dose-response as for spironolactone 25–50 mg was seen (appendix). At both doses, a correlation existed between the systolic blood pressure-lowering effect of amiloride and spironolactone (*r*^2^=0·64, p<0·0001; figure 6). No significant change in plasma sodium or eGFR occurred with either amiloride or spironolactone treatment, but plasma potassium concentrations increased from 4·02 mmol/L (95% CI 3·95–4·08) with placebo to 4·50 (4·44–4·57) with amiloride and to 4·35 (4·28–4·42) with spironolactone (both p<0·0001; appendix). No serious adverse events were recorded with either spironolactone or amiloride. Other adverse events were not systematically recorded during the open-label phase of PATHWAY-2. Adverse events recorded during double-blind treatment with amiloride are published elsewhere.^7^

## Discussion

The results of our three PATHWAY-2 mechanistic substudies show that the blood pressure response to spironolactone in resistant hypertension is predicted by ARR and by plasma renin alone, that the superior reduction of blood pressure achieved with spironolactone is associated with elimination of thoracic volume excess rather than vasodilatation, and that amiloride is similarly effective as spironolactone in reducing blood pressure in patients with resistant hypertension. We also showed that 31 (25%) of 126 patients have an inappropriately high plasma aldosterone concentration—ie, that is greater than the mean for all patients in the substudy despite a plasma renin concentration in the lowest tertile.

The PATHWAY-2 study was designed to test the hypothesis that resistant hypertension is a salt-retaining state, and that the best treatment is additional diuretic.^3^ The hypothesis was supported by the primary and secondary outcomes of the study. The aldosterone antagonist spironolactone was substantially more effective at reducing blood pressure than the licensed antihypertensive drugs bisoprolol and doxazosin, and the reduction in blood pressure with spironolactone was predicted by baseline plasma renin concentrations.^3^ The study was not designed to establish whether the mechanism of diuresis (or choice of diuretic) was crucial to the outcome. However, results from the other studies from the PATHWAY programme suggested that the reduction of blood pressure after addition of spironolactone was greater than would have been achieved by increasing the dose of thiazide. In particular, the findings from PATHWAY-3 showed that doubling the dose of hydrochlorothiazide from 25 to 50 mg reduced blood pressure by 4 mm Hg less than the combination of hydrochlorothiazide 25 mg with amiloride 10 mg in patients with hypertension.^7^

The rationale for the extra measurements or intervention that we report here was to link the pathophysiology of resistant hypertension with response to treatment. This link became of particular interest when the primary outcome showed spironolactone to be substantially superior to the alternatives. Although this outcome was scarcely a complete surprise, we had not expected superiority to be exhibited across almost the entire distribution of plasma renin, with the crossover design permitting demonstration that only ten (3%) of 326 patients in PATHWAY-2 had a plasma renin concentration that predicted better blood pressure response to α blockade or β blockade than to spironolactone. It seems reasonable now to conclude that resistant hypertension, as previously suspected,^14^ is predominantly a salt-retaining state, often caused by primary or secondary aldosteronism.

One of the best measures of sodium balance is plasma renin, as shown by the dose-related, several-times increases in plasma renin caused by each of the diuretic treatments in PATHWAY-3.^7^ Definition of normal ranges for plasma renin is difficult in patients receiving multiple antihypertensive drugs—in particular the A+C+D classes required for a diagnosis of resistant hypertension, all of which increase concentrations of plasma renin.^15^ However, the median plasma renin value of 34 mU/L in PATHWAY-2 was only three times greater than the median value (11 mU/L) in the 605 patients with untreated hypertension in PATHWAY-1, who had similar blood pressure values on no treatment.^16^ Because we can compare the same patient's response to each of a diuretic drug (spironolactone; efficacy inversely correlated to renin) and RAS-blocking drug (bisoprolol; positively correlated), we can establish the point between the bottom and top of the renin distribution at which the diuretic is predicted by the plasma renin concentration to be less effective than the RAS blocker (appendix). In the patients with resistant hypertension in PATHWAY-2, this point lies at the extreme right of the renin distribution, with all but 3% of patients predicted to respond better to diuretic than RAS blocker. This finding is in striking contrast to the effects seen in the previously untreated patients of PATHWAY-1, in whom a crossover comparison of hydrochlorothiazide 25 mg and losartan 100 mg was done, and the diuretic efficacy is predicted by plasma renin concentration to exceed that of the RAS blocker in fewer than half the patients.^16^ Notably, the relatively low median plasma renin in PATHWAY-2 is not due to impaired renal clearance because the eGFR was normal. Nor was it due to excessive dietary sodium intake as the 24 h urinary sodium was typical of that seen in western Europe.^17^

Aldosterone secretion is normally suppressed when renin is low, unless there is autonomous aldosterone production—eg, from an aldosterone-producing adenoma. This aldosterone suppression is apparent during acute salt loading, in cross-sectional studies of dietary sodium intake, in some of the rare monogenic causes of hypertension, and in patients whose low-renin hypertension is due to derivatives of naturally occurring mineralocorticoids.^18^, ^19^, ^20^ Unsuppressed plasma aldosterone values raise suspicion that they are the driver of salt retention. The paucity of patients with low plasma aldosterone and low plasma renin in our study is consistent with aldosterone having a primary role in many patients with resistant hypertension.^14^, ^21^ However, none of the patients, even one whose hypertension was cured by removal of a 7 mm aldosterone-producing adenoma,^22^ exhibited the triad of spontaneous hypokalaemia, completely suppressed renin, and plasma aldosterone of more than 550 pmol/L that is currently required for diagnosis of primary aldosteronism if a suppression test (the alternative method of diagnosis) is to be avoided.^21^

The absence of this triad could be anticipated from the eligibility criteria for PATHWAY-2. Yet, despite our exclusion of patients with known or suspected secondary hypertension, even the more conservative of our estimates for primary aldosteronism frequency (10%) exceeds the prevalence of 99 (5·9%) of 1672 unselected patients with hypertension who were recruited from primary care (like most patients in PATHWAY-2), and is similar to the 126 (11·2%) of 1125 newly diagnosed patients referred to specialist clinics.^23^, ^24^ Our estimate was enabled by the recent prospective comparison of confirmatory tests for primary aldosteronism, showing that a post-captopril ARR greater than 60·94 pmol/L^−1^ per mU/L^−1^ is equivalent to a positive saline suppression test, because all patients at entry to PATHWAY-2 were already treated with maximum-tolerated doses of RAS blocker. At most, nine patients might have been excluded from PATHWAY-2 if entry had (implausibly) required two ARR estimations in all screened patients and follow-up confirmatory tests when ARR was higher than 60·94 pmol/L^−1^ per mU/L^−1^.

However, the criteria for suspecting and diagnosing primary aldosteronism are likely to miss many of the zona glomerulosa subtype of aldosterone-producing adenomas. Their responsiveness to angiotensin and frequent gain-of-function mutations in an L-type calcium channel^25^, ^26^ imply that their aldosterone secretion might be reduced by RAS or calcium blockers sufficiently to decrease to less than guideline thresholds, but insufficiently to prevent increasingly severe and high-risk hypertension. Zona glomerulosa-like aldosterone-producing adenomas, unlike the classical Conn's adenoma, are more common in men than in women, are often small, and are more likely to present as resistant hypertension than a CT finding.^25^, ^26^, ^27^, ^28^ Our post-hoc finding of sex difference in the aldosterone–renin association is therefore notable in this context. The same is true of unilateral or bilateral hyperplasia, the pathological basis on which is increasingly recognised as clusters of aldosterone synthase-rich zona glomerulosa cells. These clusters have a several-times higher expression of adrenocorticotropic hormone receptors than adjacent zona glomerulosa, and many clusters have the same somatic mutations as are found in zona glomerulosa-like adenomas.^29^

We have therefore reported an analysis independent of ARR, which points to a substantially higher upper estimate for prevalence of primary aldosteronism in patients with resistant hypertension than the 10% estimated by ARR. Acute changes in aldosterone concentrations can result in dramatic changes in urine sodium-to-potassium ratio.^30^ At a steady state, these are less apparent, but they contribute to an index based on the SUSPPUP ratio that has only slightly lower accuracy than ARR itself in detecting patients with proven primary aldosteronism.^13^ The correlation of blood pressure response to spironolactone but not to the other drugs in PATHWAY-2 offers some independent support for the sodium-to-potassium or SUSPPUP ratio as a diagnostic threshold. The 48 (38%) of 126 PATHWAY-2 substudy patients who had a ratio greater than the published threshold^13^ of 5·3 (mmol L^−1^)^−1^ is similar to the proportion of unselected patients with hypertension who have an apparently normal ARR but develop acute hyperaldosteronism on stress (eg, treadmill test) or ultra-low dose (30 ng) of adrenocorticotropic hormone.^31^ In one study,^32^ the SUSPPUP threshold of 5·3 (mmol L^−1^)^−1^ had lower accuracy; however, some variation is unsurprising because ARR itself has no single, universally agreed threshold.^21^

What practical role, then, do we now envisage for the assessments and interventions reported here? The non-invasive haemodynamic measurements were previously proposed as a device for selecting the best treatment in resistant hypertension.^10^ However, we did not find the measurements to be useful for prediction, perhaps because only one drug was the best in the large majority of patients. Of greater practical value might be our finding that amiloride seems similarly effective to spironolactone in the treatment of resistant hypertension. The efficacy and tolerability of amiloride when used in adequate doses are supported by our randomised comparison of amiloride 10–20 mg with hydrochlorothiazide 25–50 mg in PATHWAY-3.^7^ Although spironolactone was well tolerated over 12 weeks, gynaecomastia is a concern with longer-term treatment. Retrospective reports that its anti-androgen activity is associated with lower incidence of carcinoma of the prostate might persuade some patients to tolerate the gynaecomastia.^33^, ^34^ Amiloride could now be considered an alternative for patients in whom spironolactone is not tolerated.

As for the increased awareness of primary aldosteronism as a potentially preventable or curable cause of resistant hypertension, possible investigations and treatments in selected centres and patients will probably vary greatly, as will what can be more widely recommended on the basis of available clinical and health economic evidence. Prospective evaluation of sodium-to-potassium ratios might allow their use as an initial screening filter. Management of primary aldosteronism is rapidly changing as less invasive modalities become available for diagnosis and treatment of functional adenomas.^35^

Our study has some limitations. By design, only some of the patients in PATHWAY-2 participated in the various substudies. However, baseline characteristics were similar between the cohorts. Prospective power was calculated for the primary outcome in PATHWAY-2, but not for the individual substudies, which were intended to allow prespecified, secondary mechanistic analyses that might help to explain the results of the main study. In our favour, the size of the study proved much larger than necessary to detect the primary outcome, having been powered to detect a difference of only 3 mm Hg between spironolactone and other drugs, at an α of 0·003;^3^, ^6^ indeed, post-hoc analysis of the first cycle of treatment showed the superiority of spironolactone in just 80 patients.^3^ Urine aldosterone measurements might have helped to confirm a high prevalence of primary aldosteronism. Finally, the similarity and correlation of the amiloride responses with those previously measured on spironolactone add qualified support for use of open-label treatment to simplify and encourage complex rotation studies,^6^ but do not guarantee that the two potassium-sparing diuretics are interchangeable. The finding that only the spironolactone response correlated with SUSPPUP might point to spironolactone as the more effective drug when aldosterone is the cause of sodium retention, and amiloride when the cause is clearly downstream.

We conclude that the mineralocorticoid receptor antagonist spironolactone is an effective treatment of resistant hypertension because resistant hypertension is commonly a salt-retaining condition probably due to inappropriate aldosterone secretion. Amiloride seems to be an effective, well tolerated alternative to spironolactone. Finally, our findings should encourage debate about whether thresholds for diagnosis of primary aldosteronism should be reconsidered in patients presenting with resistant hypertension, and about the possibility of earlier diagnosis of primary aldosteronism to prevent development of resistant hypertension.
