# Correction to *Lancet Diabetes Endocrinol* 2018; 6: 464–75

**DOI:** 10.1016/S2213-8587(18)30211-0

**Published:** 2018-08

*Williams, B, MacDonald, TM, Morant, SV, et al. Endocrine and haemodynamic changes in resistant hypertension, and blood pressure responses to spironolactone or amiloride: the PATHWAY-2 mechanisms substudies.* Lancet Diabetes Endocrinol *2018; **6:** 464–75*—In this Article, the unit for aldosterone-to-renin ratio (ARR) was incorrect or missing in several places in the text and in figure 3. The unit for ARR should be pmol/mU. This correction has been made to the online version as of July 24, 2018.

