# Supplementary material for: Endocrine and haemodynamic changes in resistant hypertension, and blood pressure responses to spironolactone or amiloride: the PATHWAY-2 mechanisms substudies
Source: Lancet Diabetes Endocrinol. 2018 Jun;6(6):464–75. doi: 10.1016/S2213-8587(18)30071-8 (PMC5966620; doi:10.1016/S2213-8587(18)30071-8)

THE LANCET
Diabetes & Endocrinology

Supplementary appendix

This appendix formed part of the original submission and has been peer reviewed. We post it as supplied by the authors.

Supplement to: Williams B, MacDonald TM, Morant SV, et al, for The British Hypertension Society programme of Prevention And Treatment of Hypertension With Algorithm based Therapy (PATHWAY) Study Group. Endocrine and haemodynamic changes in resistant hypertension, and blood pressure responses to spironolactone or amiloride: the PATHWAY-2 mechanisms substudies. *Lancet Diabetes Endocrinol* 2018; published online April 11. http://dx.doi.org/10.1016/S2213-8587(18)30071-8.

On-Line Appendix

Pathway Studies Investigators

1. Anne Schumann, Jo Helmy, Carmela Maniero, Timothy J Burton, Ursula Quinn, Lorraine Hobbs, Jo Palmer (Addenbrooke's, Cambridge, UK)
2. David Collier, Nirmala Markandu, Manish Saxena, Anne Zak, Enamuna Enobakhare (William Harvey Institute, QMUL, London, UK)
3. Judith Mackay, Simon A McG Thom, Candida Coughlan, (Imperial College Health Care NHS Trust, London, UK)
4. Alison R McGinnis, Evelyn Findlay (Ninewells Hospital & Medical School, Dundee, UK)
5. Adrian G Stanley, Gerry P McCann, Christobelle White, Peter Lacy, Caroline J. Gardiner-Hill, Sheraz Nazir, Pankaj Gupta. (Glenfield Hospital, Leicester, UK)
6. Scott Muir, Linsay McCallum (Glasgow Cardiovascular Research centre, Glasgow, UK)
7. Vanessa Melville, Iain M MacIntyre (University of Edinburgh Western General Hospital, Edinburgh, UK)
8. Handrean Soran, See Kwok, Karthirani Balakrishnan. (Old Saint Mary's Hospital, Manchester, UK)
9. Richard D'Souza (Exeter Hospital, Exeter, UK)
10. Richard Hobbs, Rachel Iles. (Primary Care Clinical Sciences University Birmingham, Birmingham, UK)
11. Khin Swe Myint (Clinical Research and trials Unit, University of East Anglia, Norwich, UK)
12. John Cannon, Sue Hood (Ixworth GP Practice, Ixworth, UK)
13. Krzysztof Rutkowski, Andrew Webb (Cardiovascular Medicine & Diabetes, King's College London, London, UK)
14. Una Martin (University Hospital Birmingham, Birmingham, UK)
15. Sharon Kean, Robbie Wilson, Richard Papworth (Robertson Centre for Biostatistics and Glasgow CTU, Glasgow, UK)

On-line Appendix Tables

Appendix Table 1

The data for all haemodynamic parameters.

	Stroke index (mL/beat/m^2)	Adjusted for baseline	Change from baseline	p-value
Means	Spironolactone	40.2 (38.5,41.9)	-1.6 (-3.3,0.1)	0.063
	Doxazosin	42.3 (40.6,44.0)	0.5 (-1.2,2.2)	0.601
	Bisoprolol	48.4 (46.7,50.0)	6.5 (4.9,8.2)	<.001
	Placebo	42.7 (41.0,44.4)	0.9 (-0.8,2.5)	0.318
	Overall p-value for treatment differences	<.001	<.001	
Mean differences	Spironolactone vs Doxazosin	-2.04 (-3.40,-0.68)		0.003
	Spironolactone vs Bisoprolol	-8.14 (-9.49,-6.78)		<.001
	Spironolactone vs Placebo	-2.45 (-3.80,-1.10)		<.001
	Doxazosin vs Bisoprolol	-6.09 (-7.47,-4.72)		<.001
	Doxazosin vs Placebo	-0.41 (-1.77,0.96)		0.560
	Bisoprolol vs Placebo	5.69 (4.34,7.03)		<.001

	Cardiac index (L/min/m^2)	Adjusted for baseline	Change from baseline	p-value
Means	Spironolactone	2.90 (2.79,3.00)	-0.05 (-0.16,0.06)	0.389
	Doxazosin	2.96 (2.85,3.07)	0.02 (-0.09,0.13)	0.705
	Bisoprolol	2.77 (2.66,2.88)	-0.17 (-0.28,-0.07)	0.002
	Placebo	2.98 (2.87,3.09)	0.04 (-0.07,0.15)	0.479
	Overall p-value for treatment differences	<.001	<.001	
Mean differences	Spironolactone vs Doxazosin	-0.069 (-0.158,0.021)		0.131
	Spironolactone vs Bisoprolol	0.127 (0.039,0.216)		0.005
	Spironolactone vs Placebo	-0.087 (-0.176,0.001)		0.054
	Doxazosin vs Bisoprolol	0.196 (0.106,0.286)		<.001
	Doxazosin vs Placebo	-0.018 (-0.108,0.071)		0.689
	Bisoprolol vs Placebo	-0.214 (-0.303,-0.126)		<.001

Vascular resistance index (dyne*sec/cm^5/m^2))		Adjusted for baseline	Change from baseline	p-value
Means	Spironolactone	2656 (2519,2793)	-360 (-497,-223)	<.001
	Doxazosin	2726 (2587,2865)	-291 (-430,-151)	<.001
	Bisoprolol	2781 (2643,2918)	-236 (-374,-99)	<.001
	Placebo	2806 (2667,2944)	-211 (-349,-73)	0.003
	Overall p-value for treatment differences	0.066	0.066	
Mean differences	Spironolactone vs Doxazosin	-69.8 (-188.8,49.1)		0.249
	Spironolactone vs Bisoprolol	-124.2 (-242.1,-6.2)		0.039
	Spironolactone vs Placebo	-149.1 (-267.1,-31.2)		0.013
	Doxazosin vs Bisoprolol	-54.4 (-174.3,65.5)		0.373
	Doxazosin vs Placebo	-79.3 (-198.7,40.1)		0.192
	Bisoprolol vs Placebo	-25.0 (-142.8,92.9)		0.678

Thoracic fluid index(1/kΩ/m^2)		Adjusted for baseline	Change from baseline	p-value
Means	Spironolactone	13.7 (13.4,14.1)	-1.0 (-1.3,-0.6)	<.001
	Doxazosin	15.0 (14.6,15.3)	0.3 (-0.1,0.6)	0.103
	Bisoprolol	14.4 (14.0,14.7)	-0.3 (-0.6,0.0)	0.091
	Placebo	14.4 (14.1,14.8)	-0.3 (-0.6,0.1)	0.115
	Overall p-value for treatment differences	<.001	<.001	
Mean differences	Spironolactone vs Doxazosin	-1.25 (-1.56,-0.94)		<.001
	Spironolactone vs Bisoprolol	-0.66 (-0.97,-0.35)		<.001
	Spironolactone vs Placebo	-0.68 (-0.99,-0.37)		<.001
	Doxazosin vs Bisoprolol	0.59 (0.28,0.91)		<.001
	Doxazosin vs Placebo	0.57 (0.26,0.89)		<.001
	Bisoprolol vs Placebo	-0.02 (-0.33,0.29)		0.911

Least squares means adjusted for gender, height, weight, smoking history, baseline SBP and the baseline of the outcome, from mixed models allowing for correlations between repeat measurements in each subject.

Appendix Table 2

Prediction of home SBP response to each drug by baseline parameters

		Difference in home SBP at final visit	p value
Male vs Female	Spironolactone	1.49 (-2.12,5.09)	0.420
	Doxazosin	1.34 (-1.49,4.17)	0.354
	Bisoprolol	-0.65 (-3.48,2.18)	0.652
	Placebo	0.67 (-2.14,3.49)	0.639
Age (per 10 years)	Spironolactone	-1.04 (-2.77,0.68)	0.238
	Doxazosin	-1.31 (-2.71,0.09)	0.067
	Bisoprolol	-0.01 (-1.43,1.41)	0.991
	Placebo	-0.77 (-2.14,0.59)	0.269
Weight (per 10 kg)	Spironolactone	-0.38 (-1.27,0.51)	0.408
	Doxazosin	0.03 (-0.68,0.75)	0.928
	Bisoprolol	-0.20 (-0.92,0.52)	0.587
	Placebo	0.35 (-0.35,1.04)	0.330
Current smoker	Spironolactone	1.41 (-4.55,7.38)	0.643
	Doxazosin	0.34 (-4.62,5.30)	0.893
	Bisoprolol	2.44 (-2.47,7.35)	0.331
	Placebo	-2.00 (-6.71,2.70)	0.405
Baseline HSBP (per 10 mmHg)	Spironolactone	-0.48 (-0.59,-0.37)	<.001
	Doxazosin	-0.38 (-0.47,-0.29)	<.001
	Bisoprolol	-0.22 (-0.32,-0.12)	<.001
	Placebo	-0.31 (-0.40,-0.22)	<.001
Renin (per 10 fold increase)	Spironolactone	4.90 (2.33,7.46)	<.001
	Doxazosin	-0.42 (-2.41,1.57)	0.681
	Bisoprolol	-0.89 (-3.00,1.21)	0.405
	Placebo	0.67 (-1.37,2.72)	0.519
Stroke index	Spironolactone	0.02 (-0.14,0.18)	0.824
	Doxazosin	-0.05 (-0.17,0.08)	0.481
	Bisoprolol	0.03 (-0.09,0.16)	0.601
	Placebo	0.06 (-0.07,0.19)	0.345
Cardiac index	Spironolactone	0.33 (-2.01,2.67)	0.782
	Doxazosin	0.45 (-1.46,2.37)	0.641
	Bisoprolol	-0.11 (-1.98,1.76)	0.911
	Placebo	1.18 (-0.71,3.07)	0.221

		Difference in home SBP at final visit	p value
Thoracic fluid index	Spironolactone	0.20 (-0.48,0.87)	0.565
	Doxazosin	-0.09 (-0.65,0.47)	0.749
	Bisoprolol	0.06 (-0.50,0.63)	0.823
	Placebo	-0.01 (-0.58,0.55)	0.971
Peripheral resistance index	Spironolactone	-1.35 (-3.06,0.36)	0.123
	Doxazosin	-0.73 (-2.14,0.68)	0.313
	Bisoprolol	-0.40 (-1.82,1.03)	0.587
	Placebo	-0.62 (-1.99,0.76)	0.378

Effects estimated from mixed models allowing for correlations between repeat measurements in each subject.

Appendix Table 3

Comparison of clinic blood pressure, electrolytes and renal function on amiloride and spironolactone

All values are unadjusted means (95% CI)

(a) 6 weeks

Absolute results	Amiloride	Placebo	Spironolactone	p-values for Spironolactone vs Amiloride
Dose (mg):	10		25	
SBP (mmHg) (N=143)	137.8 (135.6,139.9)	148.1 (145.9,150.3)	139.8 (137.6,142.0)	0.068
DBP (mmHg) (N=143)	81.6 (80.4,82.8)	85.5 (84.2,86.8)	82.5 (81.2,83.7)	0.202
HR (N=143)	78.1 (76.6,79.6)	77.5 (76.0,79.0)	78.2 (76.7,79.7)	0.850
Na^+ (mmol/L) (N=132)	138.4 (138.0,138.8)	140.1 (139.6,140.5)	138.3 (137.9,138.8)	0.898
K^+ (mmol/L) (N=132)	4.50 (4.44,4.57)	4.02 (3.95,4.08)	4.35 (4.28,4.42)	<.001
eGFR (mls/min) (N=65)	88.0 (83.5,92.5)	94.5 (89.7,99.3)	91.5 (86.9,96.1)	0.203
Change from baseline				
SBP (mmHg) (N=143)	-20.4 (-22.5,-18.3)	-10.0 (-12.3,-7.8)	-18.3 (-20.5,-16.2)	0.068
DBP (mmHg) (N=143)	-9.4 (-10.6,-8.2)	-5.5 (-6.7,-4.2)	-8.5 (-9.8,-7.3)	0.201
HR (N=143)	0.5 (-1.0,2.0)	-0.1 (-1.6,1.4)	0.6 (-0.8,2.1)	0.850
Na^+ (mmol/L) (N=132)	-1.8 (-2.2,-1.4)	-0.1 (-0.5,0.4)	-1.8 (-2.2,-1.4)	0.898
K^+ (mmol/L) (N=132)	0.50 (0.43,0.57)	0.01 (-0.05,0.08)	0.35 (0.28,0.41)	0.00006
eGFR (mls/min) (N=65)	-9.4 (-13.9,-4.8)	-2.8 (-7.6,1.9)	-5.8 (-10.4,-1.2)	0.203

(b) 6 and 12 weeks, for patients who increased the dose of amiloride from 10 mg to 20 mg at 6 weeks

Absolute results	Amiloride		Placebo	Spironolactone		P value for Spiro vs Amil
Dose (mg):	10	20		25	50	
SBP (N=47)	143.0 (139.7,146.3)	140.8 (137.5,144.1)	148.5 (145.1,151.9)	141.5 (138.2,144.8)	139.0 (135.6,142.5)	0.255
DBP (N=47)	85.3 (83.2,87.4)	83.1 (81.0,85.2)	87.6 (85.4,89.7)	85.5 (83.3,87.6)	83.5 (81.3,85.7)	0.733
HR (N=47)	77.2 (74.6,79.7)	77.0 (74.5,79.6)	78.2 (75.6,80.8)	77.1 (74.6,79.7)	78.2 (75.6,80.8)	0.548
Na^+ (N=44)	138.4 (137.7,139.1)	137.6 (136.9,138.3)	139.8 (139.1,140.5)	139.0 (138.3,139.7)	137.7 (136.9,138.4)	0.155
K^+ (N=44)	4.50 (4.41,4.59)	4.54 (4.45,4.63)	3.99 (3.90,4.08)	4.33 (4.24,4.42)	4.54 (4.45,4.63)	0.029
eGFR (N=21)	94.1 (86.7,101.6)	92.7 (85.7,99.7)	94.1 (87.1,101.1)	94.2 (87.2,101.2)	97.1 (89.8,104.3)	0.536
Change from baseline						
SBP (N=47)	-14.7 (-18.0,-11.4)	-16.9 (-20.2,-13.6)	-9.1 (-12.5,-5.8)	-16.2 (-19.5,-12.8)	-18.6 (-22.0,-15.2)	0.255
DBP (N=47)	-6.8 (-8.9,-4.7)	-9.0 (-11.1,-6.9)	-4.6 (-6.7,-2.4)	-6.7 (-8.8,-4.5)	-8.6 (-10.8,-6.4)	0.733
HR (N=47)	0.3 (-2.3,2.8)	0.1 (-2.4,2.7)	1.3 (-1.3,3.9)	0.2 (-2.3,2.8)	1.4 (-1.3,4.0)	0.548
Na^+ (N=44)	-1.7 (-2.4,-1.0)	-2.5 (-3.2,-1.8)	-0.2 (-0.9,0.5)	-1.1 (-1.8,-0.4)	-2.4 (-3.1,-1.6)	0.155
K^+ (N=44)	0.49 (0.40,0.58)	0.52 (0.43,0.61)	-0.02 (-0.11,0.07)	0.32 (0.23,0.41)	0.52 (0.43,0.62)	0.029
eGFR (N=21)	-4.7 (-12.1,2.8)	-6.1 (-13.1,0.9)	-4.7 (-11.7,2.3)	-4.6 (-11.7,2.4)	-1.7 (-9.0,5.5)	0.536

(c) Average of 6 and 12 weeks

Absolute results	Amiloride	Placebo	Spironolactone	p-values for Spironolactone vs Amiloride
SBP (mmHg) (N=146)	136.0 (133.5,138.6)	147.3 (144.8,149.7)	136.5 (134.0,138.9)	0.710
DBP (mmHg) (N=146)	80.1 (78.7,81.6)	84.7 (83.3,86.1)	79.9 (78.5,81.2)	0.697
HR (N=146)	79.2 (77.6,80.8)	77.4 (75.9,79.0)	78.9 (77.4,80.5)	0.761
Na^+ (mmol/L) (N=137)	138.2 (137.8,138.7)	140.0 (139.6,140.4)	137.8 (137.4,138.3)	0.0673
K^+ (mmol/L) (N=136)	4.49 (4.41,4.57)	4.02 (3.94,4.10)	4.44 (4.36,4.52)	0.207
eGFR (mls/min) (N=77)	85.4 (80.4,90.5)	94.0 (89.1,98.9)	88.7 (83.9,93.4)	0.240
Change from baseline				
SBP (mmHg) (N=146)	-22.2 (-24.7,-19.7)	-11.0 (-13.4,-8.5)	-21.8 (-24.2,-19.3)	0.710
DBP (mmHg) (N=146)	-10.4 (-11.9,-9.0)	-5.9 (-7.3,-4.5)	-10.7 (-12.1,-9.3)	0.697
HR (N=146)	1.6 (-0.1,3.2)	-0.2 (-1.7,1.4)	1.3 (-0.2,2.9)	0.761
Na^+ (mmol/L) (N=137)	-2.0 (-2.4,-1.5)	-0.2 (-0.6,0.2)	-2.4 (-2.8,-1.9)	0.0673
K^+ (mmol/L) (N=136)	0.50 (0.42,0.58)	0.03 (-0.05,0.11)	0.45 (0.37,0.53)	0.207
eGFR (mls/min) (N=77)	-12.7 (-17.7,-7.6)	-4.1 (-9.0,0.8)	-9.4 (-14.2,-4.7)	0.240

On-line Appendix Figures

Appendix Figure 1

Relationship between baseline plasma renin, aldosterone or the ARR and the BP response to placebo, doxazosin or bisoprolol. Simple linear regressions with the independent variable on a log scale.

(a) Placebo

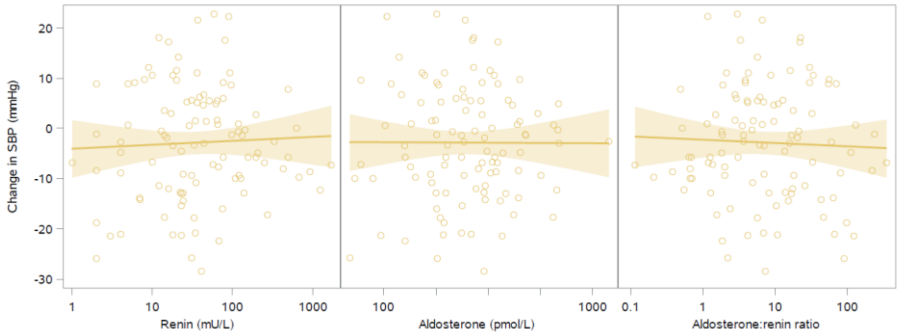

(b) Bisoprolol

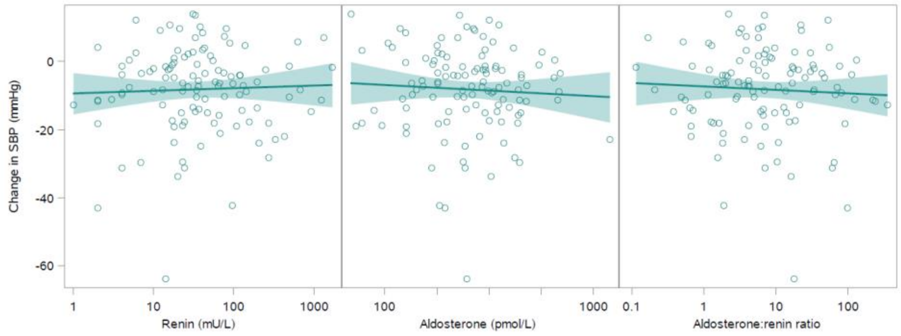

(c) Doxazosin

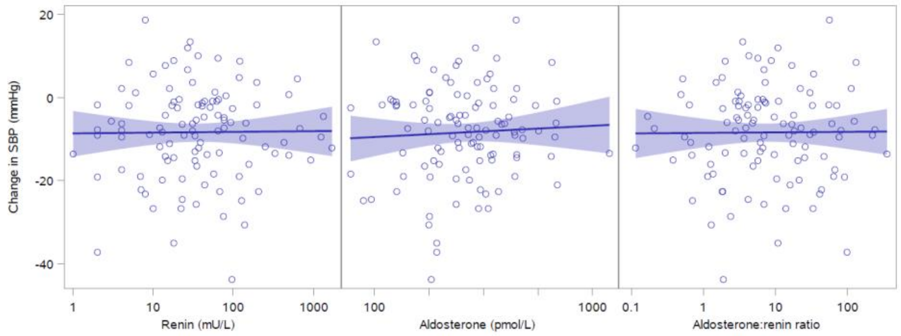

Simple linear regression with the independent variable on a log scale.

Appendix Figure 2

Gender difference in relationship of plasma aldosterone and plasma renin

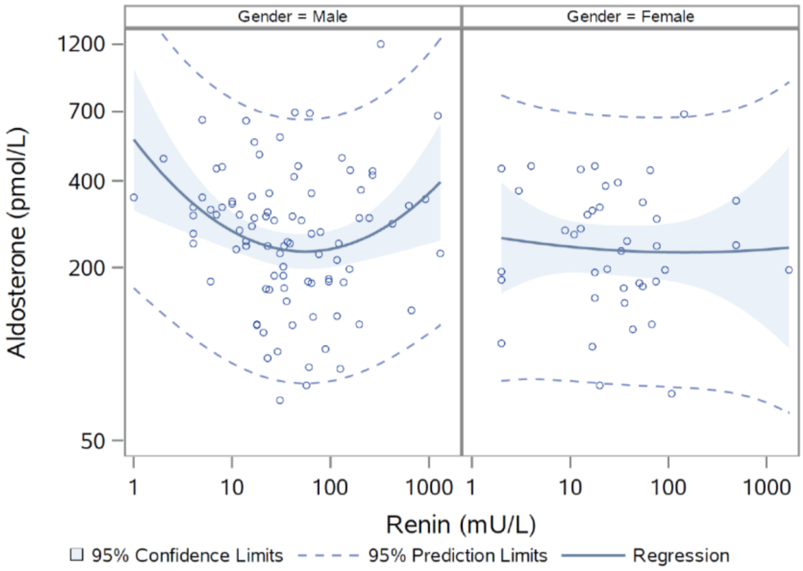

Regression equations are (y=log(aldosterone), x=log(renin)): Men $y=2.75-0.446x+0.430x^2$, $r^2=0.090$, $p=0.016$; Women $y=2.42-0.062x+0.014x^2$, $r^2=0.005$, $p=0.87$

Appendix Figure 3

Prediction of blood pressure response to spironolactone by SUSPPUP (index of Na^+/K^+ clearance)

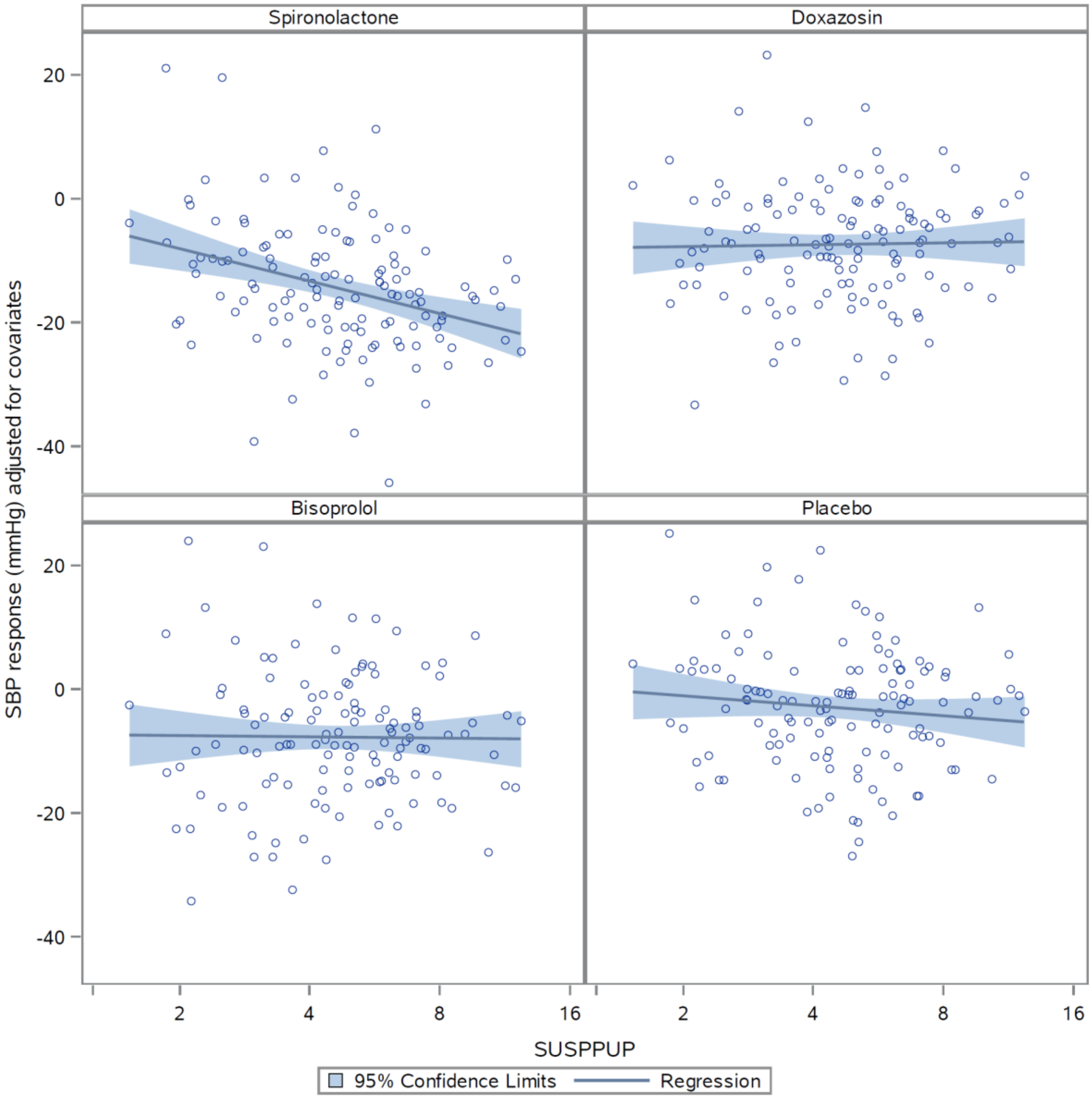

SBP response with adjustments for baseline SBP, gender, age, height, weight and smoking history estimated from mixed models allowing for correlations between repeat measurements in each subject.

	r^2	P value
Spironolactone	0.07	0.0004
Doxazosin	0.0003	0.83
Bisoprolol	0.0001	0.90
Placebo	0.01	0.27

Appendix Figure 4

Changes in body weight and comparison with similar changes in thoracic fluid.

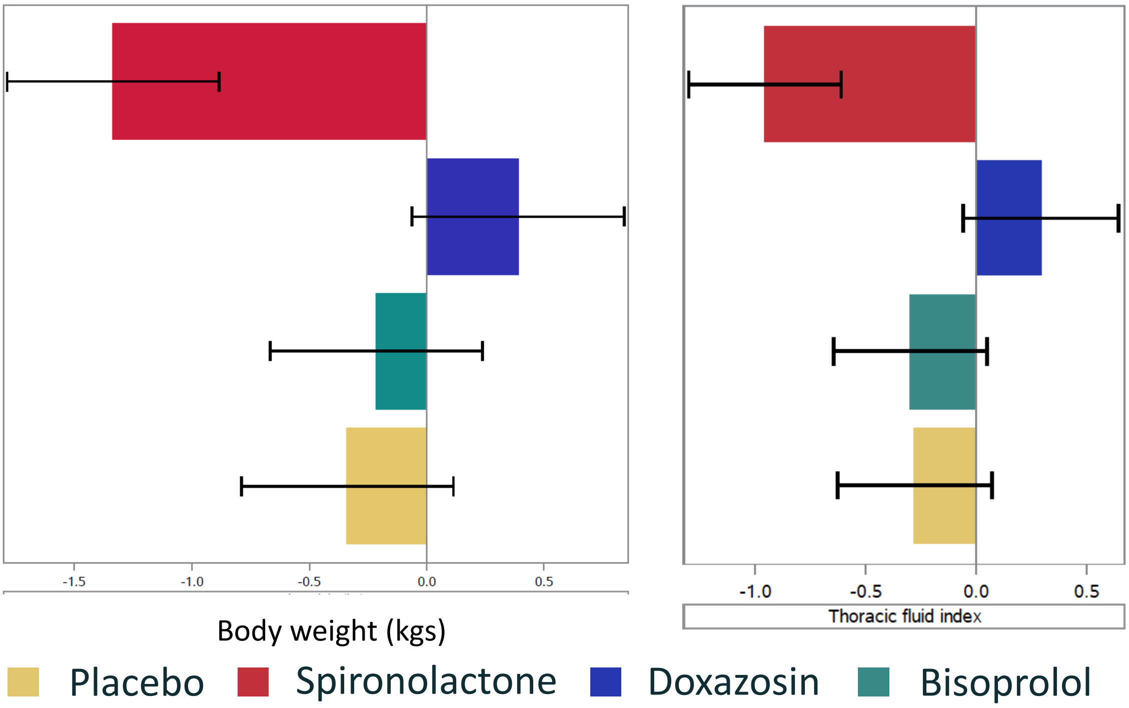

Spironolactone vs Doxazosin	-1.73 (-1.95,-1.50)	<.001
Spironolactone vs Bisoprolol	-1.12 (-1.35,-0.90)	<.001
Spironolactone vs Placebo	-1.00 (-1.22,-0.77)	<.001
Doxazosin vs Bisoprolol	0.60 (0.38,0.83)	<.001
Doxazosin vs Placebo	0.73 (0.50,0.95)	<.001

Least squares means adjusted for gender, height, weight, smoking history, baseline SBP and the baseline of the outcome, from mixed models allowing for correlations between repeat measurements in each subject.

Appendix Figure 5

Dose-response for clinic blood pressure measured on each drug in the 47 patients who up-titrated from amiloride 10 mg to amiloride 20 mg.

Values are unadjusted means for both the lower doses (hatched columns) and higher doses (solid columns).

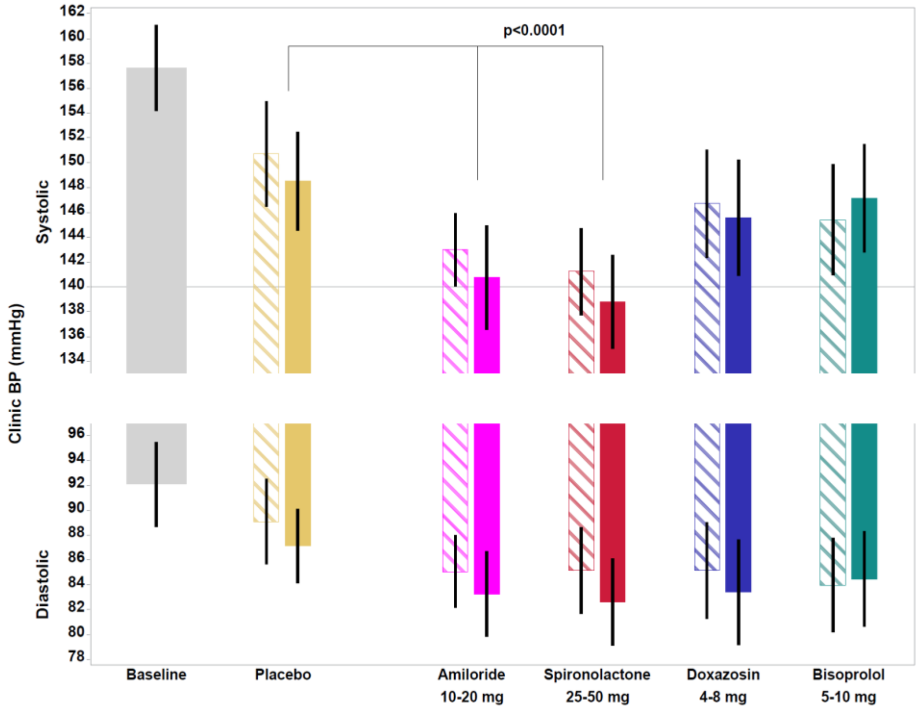

Appendix Figure 6

Difference in crossover points for delta SBP vs renin in patients with resistant hypertension (PATHWAY-2) compared to previously untreated patients (PATHWAY-1)

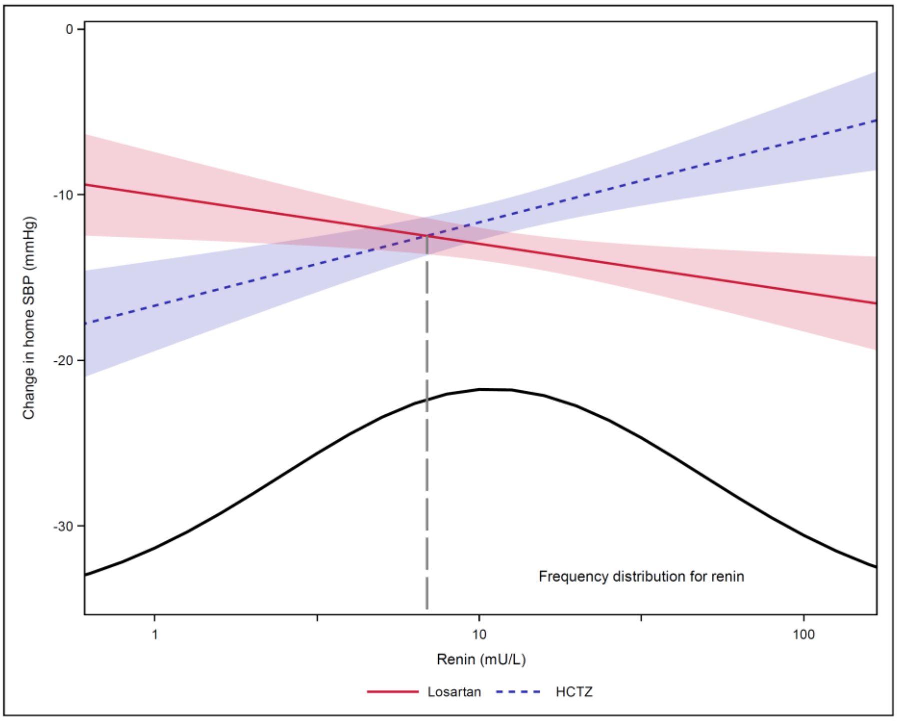

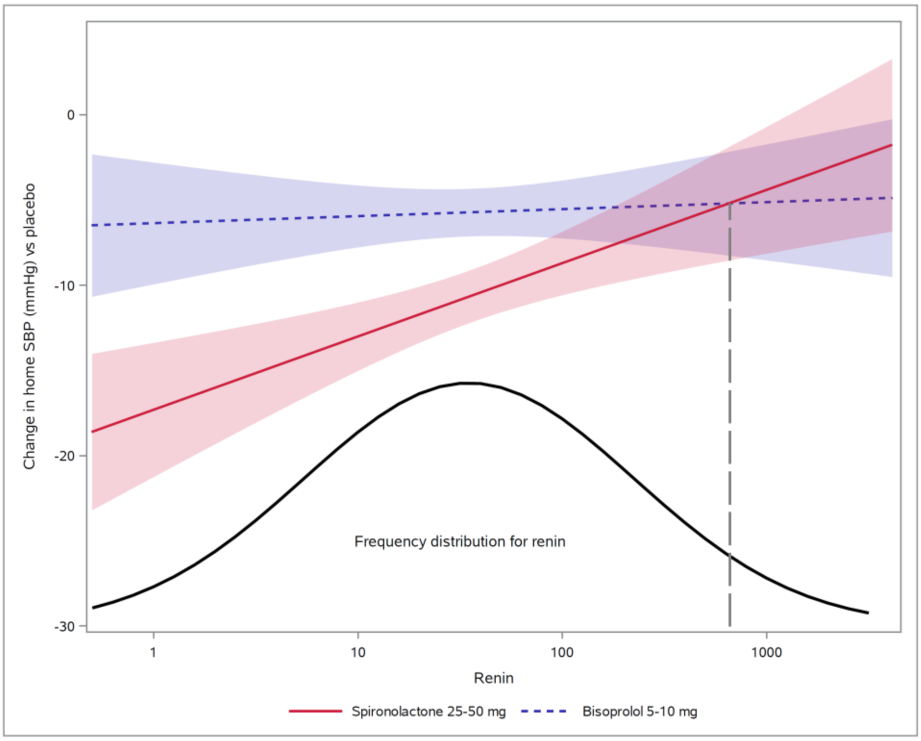

Supplement: Supplementary appendix [file mmc1.pdf]
